# P-1119. The role of Carbapenemase gene detection and synergy testing in invasive infections caused by Carbapenem resistant Enterobacterales in the pediatric population at a quaternary hospital in South India

**DOI:** 10.1093/ofid/ofae631.1306

**Published:** 2025-01-29

**Authors:** Sanjay Deshpande, Ranganathan Iyer

**Affiliations:** BAYLOR SCHOOL OF MEDICINE AND TEXAS CHILDREN'S HOSPITAL, HOUSTON, TX; RAINBOW CHILDREN'S HOSPITAL, HYDERABAD, Telangana, India

## Abstract

**Background:**

The incidence and prevalence of carbapenem-resistant *Enterobacteriaceae* (CRE) infections in children is increasing globally. However, there are limited studies that address clinical, microbiological profiles, molecular and synergy testing of these CRE infections, in this population. Our study was aimed at studying the clinical profile, microbiological and molecular diagnoses, and synergy testing of CRE bacteremia in children admitted to the intensive care unit (ICU).

**Methods:**

Medical records of children admitted to the ICU with CRE bacteraemia over a year were reviewed. Their clinical background, microbiological diagnoses, molecular profile, antibiotic resistance patterns, synergy testing, and clinical outcomes were analyzed. Carbapenemase genes were detected using Gene X pert (Cepheid) and synergy testing was carried out on those isolates with New Delhi Metalloproteinase (NDM). NDM-1 or NDM + Oxacillinase (OXA) OXA-48 genes. The results were expressed as fractional inhibitory concentration index (FICI) and the same was correlated with treatment using Ceftazidime- Avibactam+/- Aztreonam.

**Results:**

98 children with blood stream infections (40 in neonatal ICU and 58 pediatric ICU) were identified. K. *pneumoniae* accounted for a majority of BSI’s (n-34 in neonatal ICU and n- 47 in pediatric ICU) with most of them expressing the NDM gene (50%). The overall Colistin resistance across both ICU’s was (MIC > 2 ug ml) was 0.03%. Synergy to Ceftazidime-Avibactam- Aztreonam could be demonstrated in a majority of CREs which were NDM / NDM+OXA-48 producers (82.3% K. *pneumonia*e in neonatal ICU and 90% in pediatric ICU). Eight neonates (20%) and seventeen children (29%) succumbed due to other comorbidities, all of whom had NDM Klebsiella *pneumoniae* bacteraemia (n-17, 100%).

**Conclusion:**

NDM producing Klebsiella *pneumoniae* remains the commonest identified BSI in neonatal and paediatric patients in the intensive care and was isolated in all children with adverse outcomes. Infection control, antimicrobial stewardship protocols and Carbapenemase gene detection with targeted synergy and directed therapy is recommended for favorable clinical outcomes of BSI’s in the critically ill children.

**Disclosures:**

**All Authors**: No reported disclosures

